# SNaQ.jl: Improved scalability for level-1 phylogenetic network inference

**DOI:** 10.1093/bioinformatics/btag289

**Published:** 2026-05-11

**Authors:** Nathan Kolbow, Sungsik Kong, Tyler Chafin, Joshua Justison, Cécile Ané, Claudia Solís-Lemus

**Affiliations:** Wisconsin Institute for Discovery, University of Wisconsin–Madison, Madison, WI, 53706, United States; Department of Statistics, University of Wisconsin–Madison, Madison, WI, 53706, United States; Wisconsin Institute for Discovery, University of Wisconsin–Madison, Madison, WI, 53706, United States; Division of Fundamental Mathematical Science, RIKEN Center for Interdisciplinary Theoretical and Mathematical Sciences (iTHEMS), Wako, Saitama 351-0198, Japan; Department of Biological Sciences, University of Arkansas, Fayetteville, AR, 72704, United States; Tree of Life Programme, Wellcome Sanger Institute, Wellcome Trust Genome Campus, Cambridge CB10 1RQ, United Kingdom; Wisconsin Institute for Discovery, University of Wisconsin–Madison, Madison, WI, 53706, United States; Department of Statistics, University of Wisconsin–Madison, Madison, WI, 53706, United States; Wisconsin Institute for Discovery, University of Wisconsin–Madison, Madison, WI, 53706, United States; Department of Plant Pathology, University of Wisconsin–Madison, Madison, WI, 53706, United States

## Abstract

**Motivation:**

Phylogenetic networks represent complex biological scenarios that are overlooked in trees, such as hybridization and horizontal gene transfer. Although numerous methods have been developed for phylogenetic network inference, their scalability is severely limited by the computational demands of likelihood optimization and the vastness of network space. Composite (or pseudo-) likelihood approaches like SNaQ have improved computational tractability for network inference, but they remain inadequate for datasets of sizes routinely handled by tree inference methods.

**Results:**

Here, we introduce SNaQ.jl, a new standalone Julia package with the composite likelihood inference originally implemented within PhyloNetworks.jl as well as new scalability features that enhance computational efficiency through (i) parallelization of quartet likelihood calculations during composite likelihood computation, (ii) weighted random selection of quartets, and (iii) probabilistic decision-making during network search. Through a simulation study and empirical data analysis, we show that this new version of SNaQ.jl (version 1.1) improves average runtimes by up to 499% on average with no change in function parameters or method accuracy.

**Availability and implementation:**

SNaQ.jl is a new open source Julia package available at https://github.com/JuliaPhylo/SNaQ.jl.

## 1 Introduction

Phylogenetic networks depict complex biological scenarios that phylogenetic trees overlook, such as hybrid speciation, introgression, and horizontal gene transfer (Huson and Bryant 2006, Kong *et al.* 2025). Existing network inference methods that utilize full likelihood (Yu *et al.* 2014, Wen *et al.* 2016, 2018, Zhang *et al.* 2018) are not scalable enough for datasets with more than a dozen taxa, due to the high complexity and computational burden of full likelihood equations. To improve scalability, network inference methods that utilize a composite likelihood framework (sometimes referred to as pseudo-likelihood) were introduced. These methods simplify calculations by decomposing the full network into a set of smaller units (typically 4-taxon trees or networks referred to as quartets or quarnets) before computing likelihood calculations on each of these subunits. Then, the overall composite likelihood of the network is obtained as the product of the likelihoods of all quarnets. This approach has been useful in trees [e.g. MP-EST (Liu *et al.* 2010)] and networks [e.g. SNaQ (Solís-Lemus and Ané 2016), PhyNEST (Kong *et al.* 2022), PhyloNet (Yu and Nakhleh 2015, Zhu and Nakhleh 2018)] and is much faster than full likelihood methods without compromising method accuracy (Hejase and Liu 2016). Despite its computational advantages, composite likelihood estimation remains limited to inference with approximately 30 taxa, which is far below the scale of datasets typically encountered in tree analyses (e.g. One Thousand Plant Transcriptomes Initiative 2019).

Here, we introduce SNaQ.jl v1.1, a Julia package implementing the composite likelihood method for inferring level-1, identifiable, semi-directed phylogenetic networks using gene trees under the multispecies network coalescent model proposed in Solís-Lemus and Ané (2016). Additionally, we introduce the newest version of this software, version 1.1 (hereafter simply “v1.1”), a new version with improved computational efficiency via (i) parallelization of the quartet composite likelihood calculation, (ii) weighted random selection of quartets, and (iii) probabilistic decision-making during network search. The original implementation of the SNaQ method prior to these changes (hereafter simply “v1.0”) is available in SNaQ.jl for archival purposes and used here for baseline comparisons. In the following, we briefly introduce the core components of SNaQ.jl followed by the key improvements made in v1.1. Then, we present the results of a simulation study comparing the performance of each version before doing the same using an empirical dataset. Our results clearly demonstrate that SNaQ.jl significantly improves the computational efficiency of network analyses with no change in method accuracy.

## 2 Materials and methods

### 2.1 A new Julia package: SNaQ.jl

Originally developed as part of the PhyloNetworks.jl Julia package (Solís-Lemus *et al.* 2017), the composite likelihood method SNaQ (Species Networks applying Quartets; Solís-Lemus and Ané 2016) improved scalability of network inference by decomposing the full likelihood into quartet and quarnet likelihoods. In brief, for each set of four taxa, (a,b,c,d) for instance, there are three possible unrooted quartets that can be observed in a gene tree: $q_{1}=ab|cd$, $q_{2}=ac|bd$, $q_{3}=ad|bc$. SNaQ uses as input the estimated frequency of each of these quartets, denoted as quartet *concordance factors* (CFs) $X=(X_{q_{1}},X_{q_{2}},X_{q_{3}})$ (Baum 2007) in a set of gene trees across *g* loci.

Next, SNaQ searches for the network that best fits the data by traversing the space of topologically identifiable level-1 networks [see Kong *et al.* (2022) for a formal definition]. Specifically, this means that networks with 2-cycles and most 3-cycles are never proposed during network traversal [for further details see Solís-Lemus and Ané (2016)]. In order to quantify how well a given network *N* fits the data, SNaQ computes the expected concordance factors for each quarnet $q_{i}$ according to the multispecies network coalescent model (Yu *et al.* 2012, Degnan 2018). We denote expected concordance factors for a given network *N* as $\left(CF_{q_{1}},CF_{q_{2}},CF_{q_{3}}\right)$. Then, a composite likelihood function is calculated with these data as follows:


(1) $$L=\prod_{s\in S}{\left(CF_{q_{1}}\right)}^{X_{q_{1}}}{\left(CF_{q_{2}}\right)}^{X_{q_{2}}}{\left(CF_{q_{3}}\right)}^{X_{q_{3}}}$$

where *S* is the collection of all possible sets of 4 taxa.

Finally, SNaQ applies a hill-climbing algorithm to search for the network topology that maximizes the composite likelihood *L*. The algorithm explores the network space using five topological moves: (i) nearest-neighbor interchange (NNI), (ii) addition of a reticulation, (iii) changing the direction of a reticulation edge, and moving either (iv) the target, or (v) the origin of an existing reticulation edge. Note that reticulation removal is not considered as a network move because the algorithm uses strict hill-climbing and removal of a reticulation will always result in a lower (or equal) value of *L*.

The network space search begins with an initial network topology $N_{0}$ and its corresponding composite likelihood $L_{0}$. To explore potential improvements, the algorithm generates a new network topology by randomly applying one of the aforementioned five topological moves. This generates a new network $N_{1}$ with composite likelihood $L_{1}$. If $L_{1}>L_{0}$, the new topology is considered an improvement and becomes the focus of subsequent evaluations (i.e. assigning $N_{1}$ as $N_{0}$). Otherwise, $N_{1}$ is discarded and different topological moves are attempted instead. This iterative process continues until no further improvements are found after a predefined number of attempts. Additionally, to mitigate the risk of converging on local optima, SNaQ conducts multiple independent runs, by default 10, with varied starting topologies.

### 2.2 Improvements to SNaQ.jl (version 1.1)

#### 2.2.1 Computational parallelizations

SNaQ.jl v1.0 parallelizes independent runs across processors, but does not have any parallelization within individual runs. SNaQ.jl v1.1 reduces this bottleneck by parallelizing composite likelihood computations and all quartet related operations within individual runs across multiple threads.

#### 2.2.2 Quartet sampling

In SNaQ.jl v1.0, every available 4-taxon subset is used when computing the composite likelihood function. This scales quartically with the number of taxa *n* because the total number of 4-taxon sets on *n* taxa is $k=\left(\begin{array}{c} n\\ 4 \end{array}\right)=O(n^{4})$. In SNaQ.jl v1.1, we introduce the argument propQuartets. This argument takes a decimal value in (0,1] specifying the proportion of 4-taxon subsets that are utilized when computing a network’s composite likelihood. Note that propQuartets = 1 (which remains as the default) corresponds to the approach utilized in v1.0. So, if there are *k* 4-taxon subsets in the CF input table, a subset of ⌈k×propQuartets⌉ are chosen uniformly at random at the beginning of each run. These subsets are controlled by the initial seed provided to the algorithm, so results are deterministic and reproducible.

This subsampling approach has already been successfully used in phylogenetic inference (Chifman and Kubatko 2014, 2015). Once the network topology search is completed, one final round of numerical optimization is performed with all *k* CFs to attain the final network’s full log composite likelihood score.

#### 2.2.3 Topological moves using quartet weighting

In SNaQ.jl v1.0, topological moves (iii), (iv), and (v) listed above involve the random selection of a reticulation edge that acts as the focus of the topological modification or “move.” For example, in “move origin,” we select one reticulation node at random and one reticulation edge connected to this reticulation node. Then, the parent or “origin” of this reticulation edge is moved to a randomly chosen neighbor edge. This process is often wasteful as it does not consider whether the current placement of the origin was a good fit for the data.

In SNaQ.jl v1.1, we introduce the novel argument probQR, which takes a value in [0,1] and defines the probability of utilizing weighted random selection instead of simple random selection when choosing the reticulation edge at which these topological moves are performed. Note that probQR = 0 (which remains as the default) corresponds to the approach utilized in v1.0. When weighted random sampling is used, each quartet $q_{i}$ extracted from the current network is given a weight $W=\sum_{i=1}^{3}\left|X_{q_{i}}-CF_{q_{i}}\right|$ based on the absolute difference between the observed ($X_{q_{i}}$) and the expected ($CF_{q_{i}}$) concordance factors. On 4-taxon sets with high weights, the current network CFs fit the data poorly. A 4-taxon set is sampled at random using these weights, and one of the edges spanned by the associated quarnet is sampled uniformly at random to be the focus of the move in order to sample poorly fitting edges more frequently.

### 2.3 Evaluation using simulated and empirical data

#### 2.3.1 Simulations

**Figure 1:**
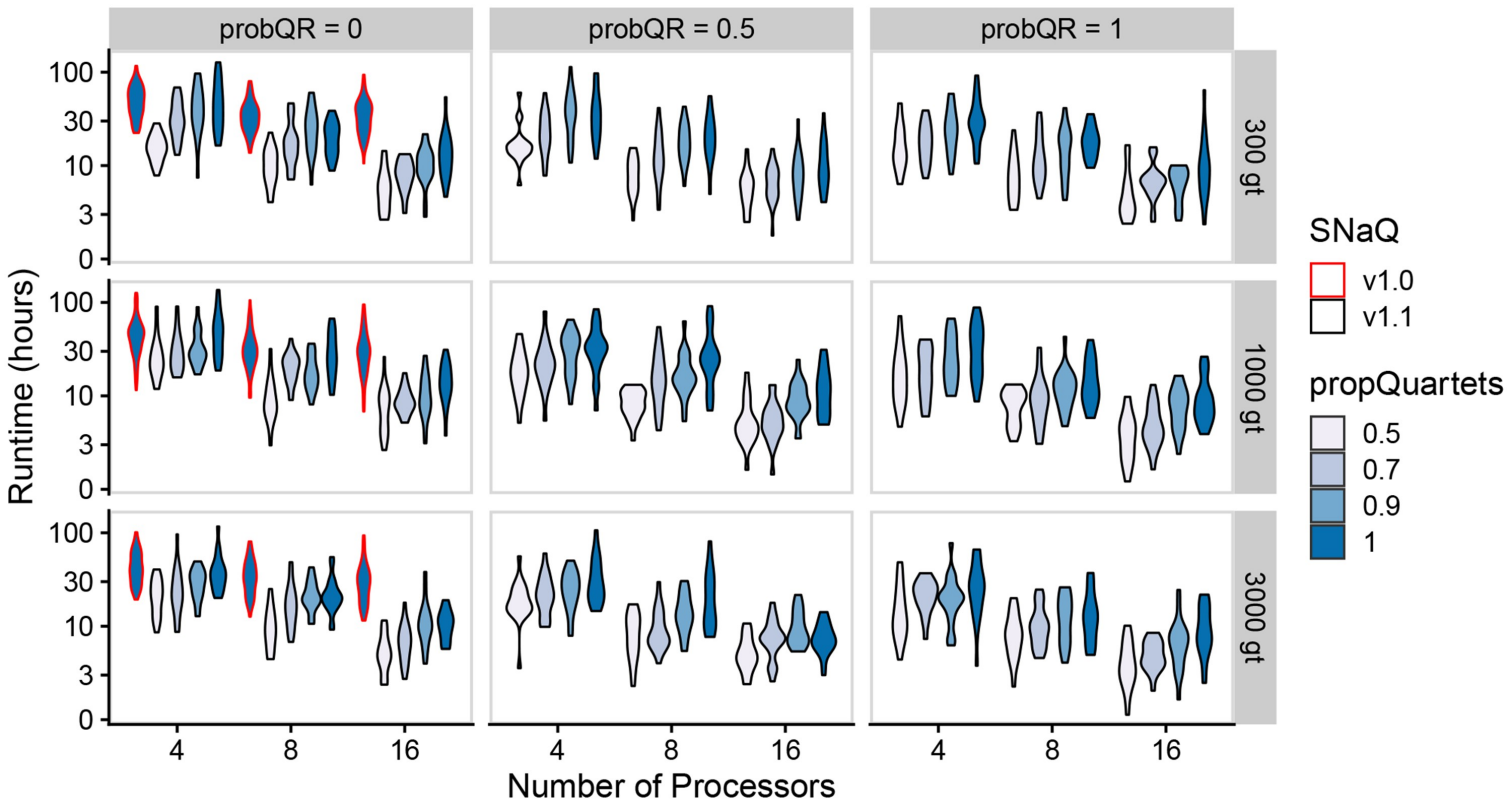
Runtimes (measured in hours and plotted on a ${\;\text{log}\;}_{10}$ scale; *y*-axis) for the network with 20 taxa and 3 reticulations. probQR, the number of input gene trees (gt), and the number of processors used was varied across simulations. SNaQ.jl v1.0 results are outlined in red while v1.1 results are outlined in black. Each violin plot covers 20 replicates.

To evaluate the performance of SNaQ.jl v1.1, we generated a set of species networks with n∈{10,20} taxa and h∈{1,3} reticulations. To do so, we first generated a species tree under a Yule process using the R package phytools (Revell 2012) for each *n*, then we added *h* reticulations onto the topologies at arbitrary positions. Three of these networks feature primarily shallow reticulations while one features deep reticulations (see Fig. 1, available as supplementary data at *Bioinformatics* online for topologies). We ensured that each network was level-1 using the R package SiPhyNetworks (Justison *et al.* 2023). To simulate low, medium, and high levels of incomplete lineage sorting, we set the average branch length in each network to 2.0, 1.0, and 0.5 respectively, excluding minor hybrid edges which had length 0. Inheritance proportions γ were 0.5 in all cases.

Using the Julia package PhyloCoalSimulations.jl (Fogg *et al.* 2023), we generated a set of g∈{300,1000,3000} gene trees for each species network. For each gene tree, we generated a multiple sequence alignment with 1000 base pairs using seq-gen (Rambaut and Grass 1997), setting the branch length scale parameter to 0.03 and base frequency of nucleotides as A=T=0.3 and C=G=0.2 under the HKY85 model (Hasegawa *et al.* 1985). These sequence alignments were then used to estimate gene trees with IQ-TREE (version 1.6.12) (Nguyen *et al.* 2015) under its automatic model selection option under Bayesian information criterion. Gene tree estimation errors (GTEEs) were computed by dividing the hardwired cluster dissimilarity (HWCD) between each estimated gene tree and its corresponding true gene tree by the maximum possible HWCD value of 2(n−3) where *n* is the number of taxa in the tree. Average GTEEs ranged across simulations from 0.21 to 0.32.

Observed CFs were calculated from the estimated gene trees and used as input for SNaQ.jl v1.0 and v1.1. Starting topologies were randomly selected from among the estimated gene trees and the true *h* was specified for each simulation. Default optimization parameters were used for both versions of SNaQ.jl, but we additionally specified probQuartets  ∈{1.0,0.9,0.7,0.5} and probQR  ∈{0,0.5,1.0} for v1.1. We computed the HWCD between the estimated and true networks using the Julia package PhyloNetworks.jl (Solís-Lemus *et al.* 2017) and recorded the runtime for each network analysis. The analyses were executed with 4, 8, and 16 processors and 2 threads per processor to assess efficiency in different computational settings. This process was repeated 100 times when n=10 and 20 times when n=20 for each set of parameters. For each replicate, new gene trees and multiple sequence alignments were simulated each time. Each replicate analysis was conducted with 10 independent SNaQ.jl runs.

Simulations were performed on a computing cluster where hardware is not uniform, but all simulations were limited to a given number of processors and 64–128 GB of memory.

#### 2.3.2 Empirical data

We repeated the analysis of 24 swordtails and platyfishes (*Xiphophorus*: Poeciliidae) from Solís-Lemus and Ané (2016) with data consisting of 1183 gene trees from Cui *et al.* (2013). Networks were inferred with h=0 to 5 reticulations where each network with h>0 used the previous best scoring network as its starting point. When h=0, a random estimated gene tree was used as the inference starting point. After obtaining these networks, a single best-fitting network was selected by applying the slope heuristic method implemented in CAPUSHE (Baudry *et al.* 2012) to the networks’ composite likelihood scores. Inference was performed with 10 processors, 2 threads per processor, and default arguments to illustrate that previous empirical results can be attained much faster as a consequence of the new parallelizations introduced in SNaQ.jl v1.1.

## 3 Results and discussion

### 3.1 Simulations

**Figure 2:**
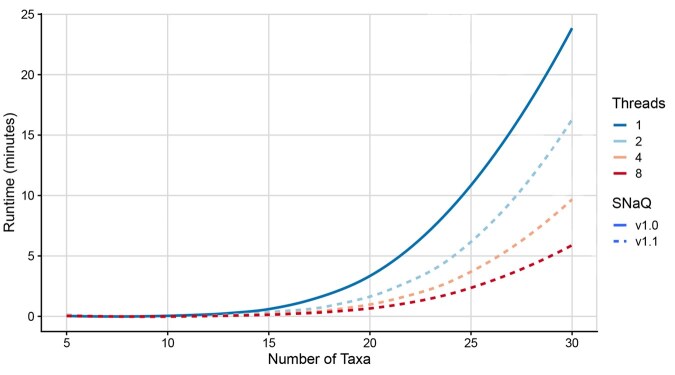
Runtimes (measured in minutes; *y*-axis) taken to optimize the parameters of individual networks using SNaQ.jl v1.0 and v1.1. v1.1 was run with 2, 4, and 8 threads, while v1.0 was only run with 1 thread. Results are averaged across replicates with 0–3 hybridizations.

As expected, we observed no noticeable difference in method accuracy between v1.1 and v1.0 when identical parameters were used. Additionally, there was no clear degradation in accuracy as propQuartets decreased (see Figs 2–5, available as supplementary data at *Bioinformatics* online), even as the number of gene trees and probQR were varied.

Next, we highlight improvements in the efficiency of SNaQ.jl v1.1 relative to v1.0 (Fig. 1 for full runs with 20 taxa and 3 hybridization events, Figs 10–12, available as supplementary data at *Bioinformatics* online for other networks; Fig. 2 for individual optimization iteration runtimes). First, SNaQ.jl v1.1 utilizes computational resources more efficiently than v1.0. For instance, runtimes for v1.0 changed very little as the number of processors increased, but runtimes for v1.1 fell drastically. This behavior occurred when method parameters were identical between v1.0 and v1.1, so this improvement is the effect of the parallelization across threads.

While we expected higher probQR values to reduce the time spent searching for networks or to improve inference accuracy, there was no noticeable improvement in either metric when probQR was changed. We suspect that higher values of probQR could be reducing the overall stochasticity of the search algorithm resulting in higher chances of being stuck in local optima. On the other hand, alternative weighting methods or different network topologies could yield different results but are outside the scope of this study. Such adaptations would be interesting to examine in future work.

The effect of propQuartets on the performance of SNaQ.jl v1.1 was clear in our simulations. Decreasing propQuartets led to significant improvements in runtime performance without no noticeable drop in accuracy. The most significant average speed improvement observed in these simulations was by a factor of 811% on the network with n=20,h=1 with 16 processors, g=1000, probQR=1, and propQuartets=0.5. Additionally, across all simulations with 16 processors, v1.1 cuts runtimes compared to v1.0 by factors of 499%, 576%, and 757% with propQuartets=1.0, 0.9, and 0.7 respectively.

When propQuartets<1, a simple random sampling approach is taken, but a more informative sampling approach, such as the weighted sampling approach of Zhang and Mirarab (2022), may lead to more robust results and enable even smaller values of propQuartets to be utilized while retaining overall method accuracy.

Lastly, the runtime improvements seen in parameter optimizations on individual networks (Fig. 2) are roughly proportional with respect to the number of threads used, indicating an efficient multi-threading implementation. It is clear, though, that while these improvements drastically improve the time taken to optimize a given network’s parameters, they do not significantly extend the size of network that can be efficiently inferred. This is especially evident when considering that larger networks naturally have larger spaces to traverse, warranting the use of more time consuming optimization routines. Thus, researchers who wish to infer drastically larger networks should continue to use more appropriate methods such as InPhyNet (Kolbow *et al.* 2025) when the primary concern is the biological accuracy of the inferred network, or Neighbor-Net (Bryant and Moulton 2004) when the primary concern is in understanding the discordance present in the set of inferred gene trees.

### 3.2 Empirical data

SNaQ.jl requires the number of hybridizations in the inferred network to be specified prior to inference. To pick the best-fitting network to the observed data, networks were inferred with h=0 to 5 reticulations and default function arguments, after which a single best-fitting network was selected by applying the model selection method implemented in the CAPUSHE R package (Baudry *et al.* 2012) to the networks’ composite likelihood scores. Then, we inferred an additional network with this selected value (h=2) and propQuartets=0.1,0.3,0.5,0.7 to assess how empirical conclusions may differ when utilizing less quartet data.

SNaQ.jl v1.1 with default function arguments took 54 h in aggregate to compute all 6 network topologies (each of which has different *h*), which is 346.2 fewer hours (14.4 fewer days) than v1.0 took originally, equating to a runtime improvement by a factor of 641%. The longest runs for v1.0 and v1.1 were 208.8 and 16.5 h, respectively, equating to an improvement in worst-case runtimes by a factor of 1165%. SNaQ.jl v1.1 with propQuartets=0.1,0.3,0.5,0.7 and h=2 took 3.6, 8.2, 6.6, and 17.35 h to infer, respectively, equating to runtime improvements by factors of 339%, 93%, 139%, and −9% compared to v1.1 with propQuartets=1.0, respectively.

Next, we examine topological differences in the networks inferred by v1.0 and v1.1 (see Figs 13–18, available as supplementary data at *Bioinformatics* online). Networks with h=0 were identical when inferred with both versions and were also identical to the total evidence tree in Cui *et al.* (2013). Networks with h=1 contained the same hybridization event and differed only in a small change in the placement of *X. milleri*. Both networks with h=2 identified this hybridization as well. Additionally, they identified the same target for their second hybridization (*X. helleri*) but slightly different origins (*X. clemenciae* versus its ancestor). Similarly, networks with h=3 and h=4 contained these two identical hybridizations but differed in the placement of their remaining hybridizations. The difference in composite log likelihood scores between v1.0 and v1.1 for h=2,3,4 were each small (≤3%). Finally, networks with h=5 had the same inferred topologies. Inferred inheritance probabilities (γ) were roughly similar between the two versions, regardless of differences in the placements of hybridizations.

Despite the similarity of these networks to those in Solís-Lemus and Ané (2016), the network inferred by SNaQ.jl v1.1 with h=1 had a drastically better composite log likelihood score than its counterpart inferred by SNaQ.jl v1.0. As a result, the best-fitting network determined by slope heuristics would be the network inferred with h=1, in contrast to Solís-Lemus and Ané (2016), where the network with h=2 was selected.

Finally, we denote the best-fitting network inferred with h=2 and propQuartets=p as $N_{p}$. Networks $N_{0.1}$ and $N_{0.7}$ had similar composite log likelihood scores to $N_{1.0}$ while $N_{0.3}$ and $N_{0.5}$ had *better* scores than $N_{1.0}$ (see Fig. 20, available as supplementary data at *Bioinformatics* online). Additionally, the topologies of these networks were all remarkably similar. Every network had the same major tree and the same reticulation from *X. xiphidium* to the northern platyfish clade. Further, the second reticulation in each network was always near the siblings *X. monticolus* and *X. clemenciae*, as well as the species *X. helleri*, but the exact location and direction of the reticulation varied.

The networks with $N_{0.3}$ and $N_{0.5}$, which had the best composite log likelihood score, estimated inheritance parameters of γ=0.43 and γ=0.32, whereas the other networks estimated γ=0.028 and γ=0.029. These differences stand out because they lead to drastically different interpretations.

Most strikingly was that $N_{0.1}$ was *identical* to $N_{1.0}$. We assumed that very low values of propQuartets would naturally lead to poorly fitting results, which is why our simulation study only went down to propQuartets=0.5, but this result clearly disproves that notion. This result is surprising, but supported by Rosas-Puchuri *et al.* (2025), who use even fewer quartets and still achieve high levels of inference accuracy [see Fig. 5 in Rosas-Puchuri *et al.* (2025)].

## 4 Conclusion

SNaQ.jl v1.1 implements parallel computing techniques to significantly improve its computational efficiency when run on a machine with multiple processing cores and threads. In our simulation study, we observed runtime improvements by 499% when comparing v1.1 to v1.0 when identical arguments were utilized with 16 processors and 2 threads per processor. Further, we observed average runtime improvements by 757% when the new optimization parameter propQuartets was reduced to 0.5. These runtime improvements come without any significant change in accuracy, indicating the v1.1 should be preferred over v1.0. This efficiency was further substantiated by re-analyzing an empirical *Xiphophorus* fish dataset with 24 taxa where runtimes improved by a similar magnitude and major findings in the original analysis were confirmed.

In addition, SNaQ.jl v1.1 implements two new parameters to the snaq! function: propQuartets and probQR. Our simulation study shows that reducing propQuartets as low as 0.5 significantly improves runtime with no significant difference in accuracy. Further, our empirical results recover identical networks when using propQuartets=1.0 and propQuartets=0.1, suggesting that even smaller subsets of quartets can lead to useful results. These results may be partially dependent on the specific network topologies analyzed here, though, so further investigation is warranted to verify the robustness of this parameter to various topological features. Additionally, future work could build on these results by identifying values of propQuartets for which SNaQ.jl’s accuracy begins to deteriorate, or by improving upon the current implementation by utilizing more sophisticated sampling strategies. On the other hand, probQR appeared to have no effect on method accuracy or runtime, so further investigation is warranted to assess why this is the case, and whether different weighted sampling approaches could yield better results.

## Supplementary Material

btag289_Supplementary_Data

## Data Availability

SNaQ.jl is a new open source Julia package available at https://github.com/JuliaPhylo/SNaQ.jl. Scripts for simulation and empirical data analyses can be found on Zenodo (https://doi.org/10.5281/zenodo.18664095). Original data used and generated in these simulations can be found on Dryad (https://doi.org/10.5061/dryad.pnvx0k721).
