# Supplementary material for: SNaQ.jl: Improved scalability for level-1 phylogenetic network inference
Source: Bioinformatics. 2026 May 11;42(6):btag289. doi: 10.1093/bioinformatics/btag289 (PMC13224965; doi:10.1093/bioinformatics/btag289)
Supplement: btag289_Supplementary_Data [file btag289_supplementary_data.pdf]

# Supporting Information

## SNaQ.jl: Improved Scalability for Phylogenetic Network Inference

Nathan Kolbow[1,2], Sungsik Kong[1,3], Tyler Chafin[4,5], Joshua Justison[1], Cécile Ané[2], and Claudia Solís-Lemus[1,6,*]

[1]Wisconsin Institute for Discovery
[2]University of Wisconsin-Madison, Department of Statistics
[3]University of Wisconsin-Madison, Department of Plant Pathology
[4]RIKEN Center for Interdisciplinary Theoretical and Mathematical Sciences, Division of Fundamental Mathematical Science
[5]Biomathematics and Statistics Scotland
[6]University of Wisconsin–Madison, Department of Plant Pathology
[*]Contact author: solislemus@wisc.edu

# Contents

**1 Simulation Study** **2**
1.1 Simulated Network Topologies . . . . . . . . . . . . . . . . . . . . . . . . . . . . . . 2
1.2 Accuracy results . . . . . . . . . . . . . . . . . . . . . . . . . . . . . . . . . . . . . 3
1.3 Runtime results . . . . . . . . . . . . . . . . . . . . . . . . . . . . . . . . . . . . . . 9

**2 Empirical Results** **11**
2.1 Inferred networks . . . . . . . . . . . . . . . . . . . . . . . . . . . . . . . . . . . . . 11
2.2 Model selection curves . . . . . . . . . . . . . . . . . . . . . . . . . . . . . . . . . . 14
2.3 Networks inferred with $h = 2$ and `propQuartets` $< 1$ . . . . . . . . . . . . . . . . . . . 15

# 1 Simulation Study

## 1.1 Simulated Network Topologies

Below are the network topologies used in the simulation study. Note that in each simulation, branch lengths were adjusted as described in the main text to simulate varying levels of incomplete lineage sorting. Specifically, to simulate low, medium, and high levels of ILS, we multiply every edge in the network by a constant such that the average branch length in the network was 2.0, 1.0, or 0.5 respectively.

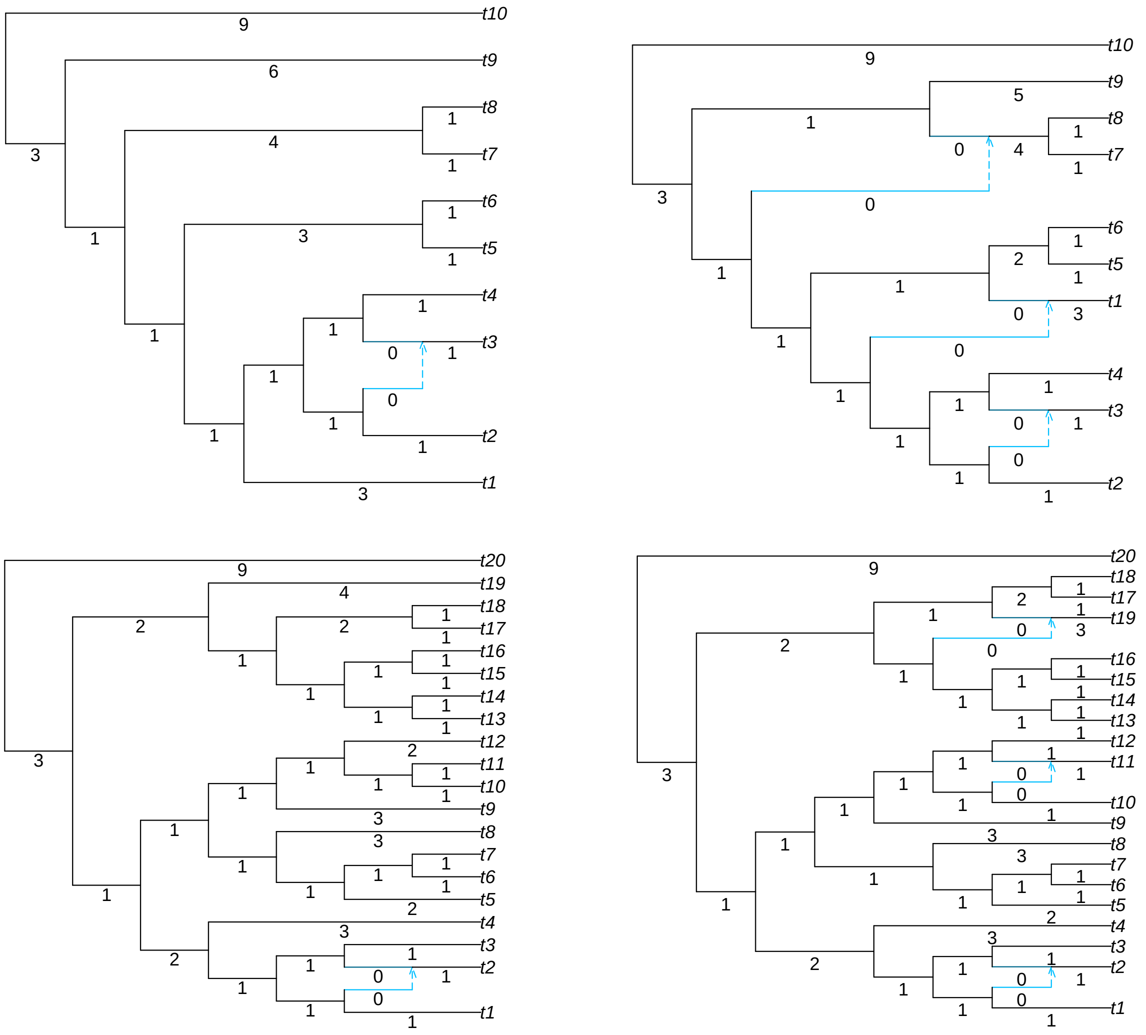


Figure S1: Topologies and edge lengths of all networks used in the simulation study. Inheritance proportions ($\gamma$, not shown) are 0.5 in all networks.

## 1.2 Accuracy results

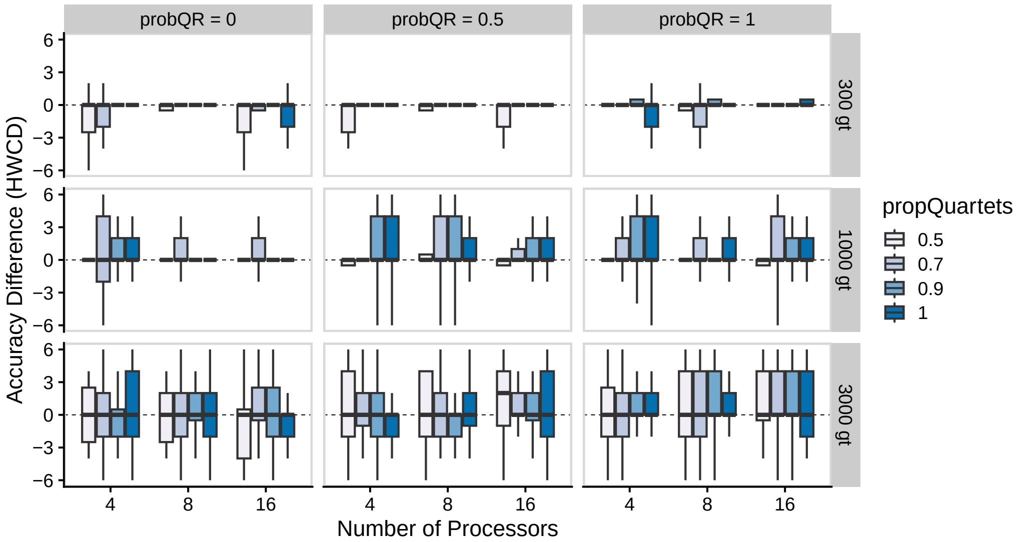


Figure S2: Differences in the accuracies of networks inferred with 10 taxa and 1 reticulation between `SNaQ.jl` v1.0 and v1.1 measured in hardwired cluster distance (HWCD) across an array of simulation parameters. Positive numbers represent v1.1 inferring a more accurate network than v1.0, while negative numbers represent the inverse, and a value of 0 represents networks with equal accuracy relative to the ground truth.

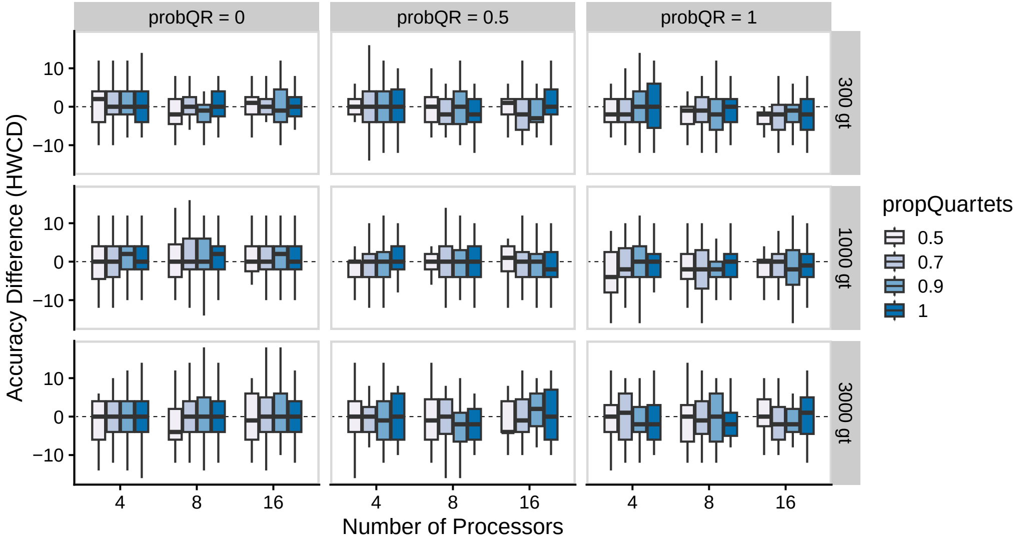


Figure S3: Accuracy results in hardwired cluster distance (HWCD) for the topology with 10 taxa and 3 reticulations across a wide array of simulation parameters. Violin plots outlined in red represent results for `SNaQ.jl` version 1.0, whereas all others represent results for version 1.1.

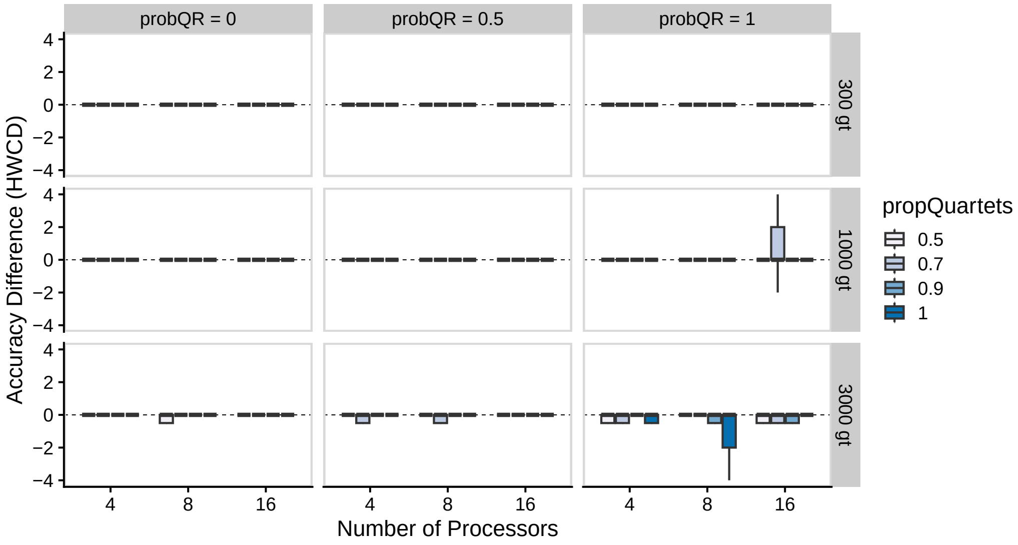


Figure S4: Differences in the accuracies of networks inferred with 20 taxa and 1 reticulation between `SNaQ.jl` v1.0 and v1.1 measured in hardwired cluster distance (HWCD) across an array of simulation parameters. Positive numbers represent v1.1 inferring a more accurate network than v1.0, while negative numbers represent the inverse, and a value of 0 represents networks with equal accuracy relative to the ground truth. This network was particularly easy for `SNaQ.jl` v1.0 and v1.1 to achieve a HWCD value of exactly 4, but very difficult to improve past that point, which is why most values here are exactly 0 (see also Fig S8).

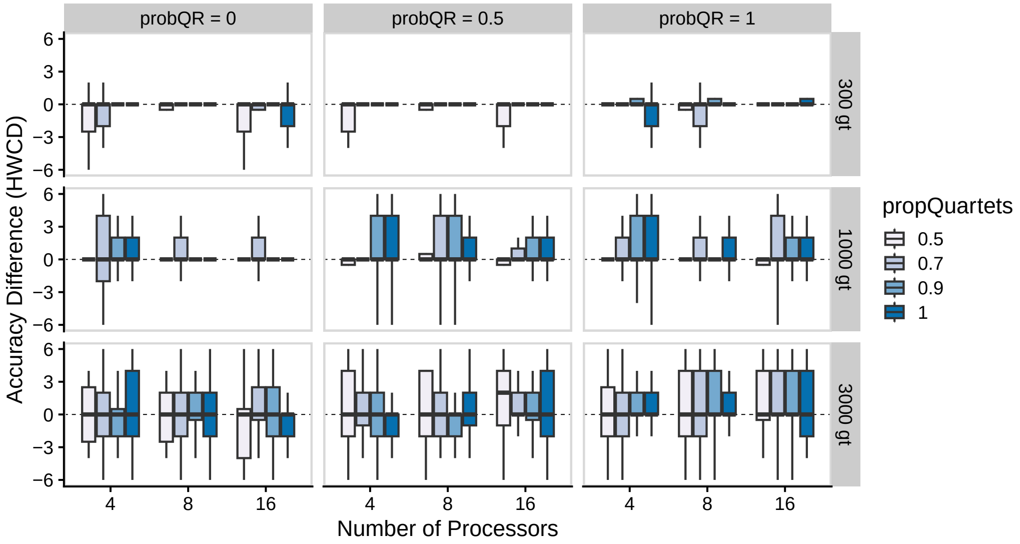


Figure S5: Differences in the accuracies of networks inferred with 20 taxa and 3 reticulation between `SNaQ.jl` v1.0 and v1.1 measured in hardwired cluster distance (HWCD) across an array of simulation parameters. Positive numbers represent v1.1 inferring a more accurate network than v1.0, while negative numbers represent the inverse, and a value of 0 represents networks with equal accuracy relative to the ground truth.

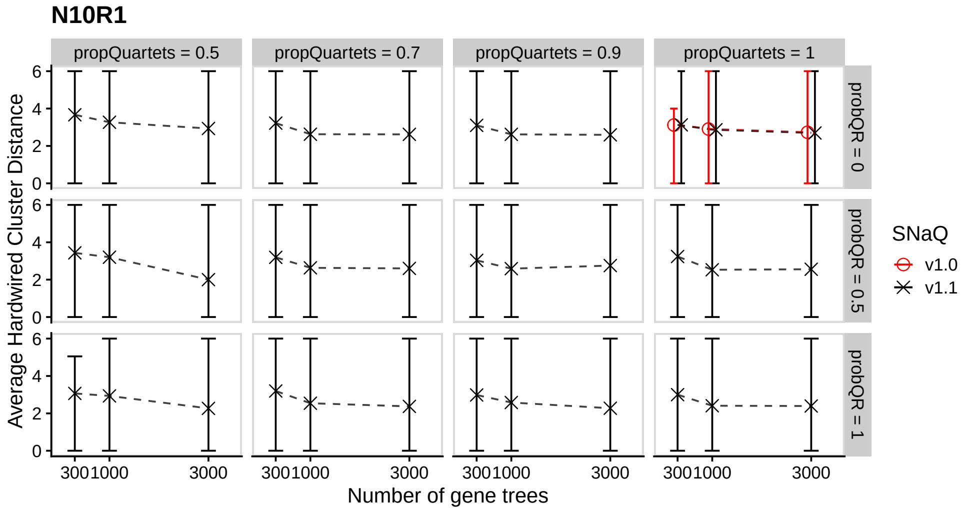


Figure S6: Accuracy results in hardwired cluster distance (HWCD) for the topology with 10 taxa and 1 reticulation relative to the number of input gene trees across a wide array of simulation parameters. Margins represent 95% empirical confidence intervals for observed accuracies, while point estimates are the mean HWCDs for each parameter combination. Simulations utilizing `SNaQ.jl` version 1.0 are shown in red, whereas those utilizing `SNaQ.jl` version 1.1 are shown in blue.

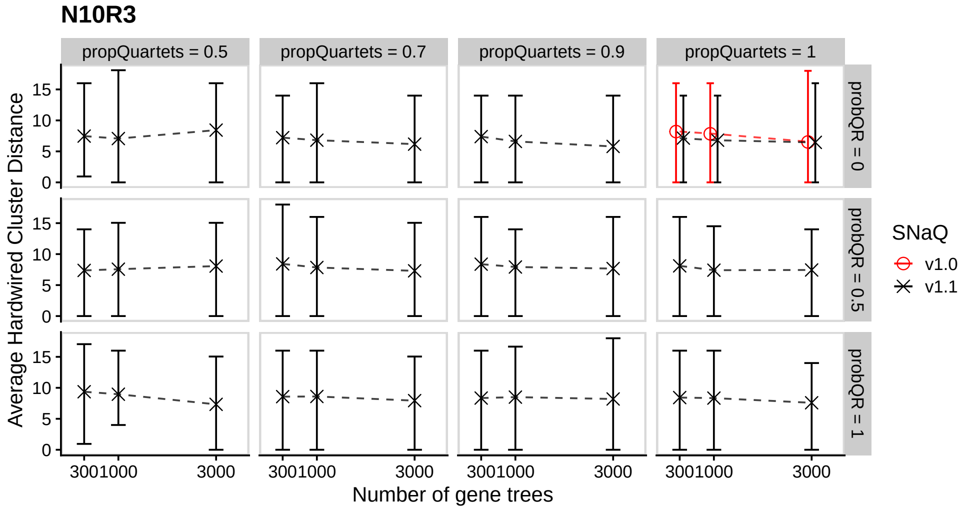


Figure S7: Accuracy results in hardwired cluster distance (HWCD) for the topology with 10 taxa and 3 reticulations relative to the number of input gene trees across a wide array of simulation parameters. Margins represent 95% empirical confidence intervals for observed accuracies, while point estimates are the mean HWCDs for each parameter combination. Simulations utilizing `SNaQ.jl` version 1.0 are shown in red, whereas those utilizing `SNaQ.jl` version 1.1 are shown in blue.

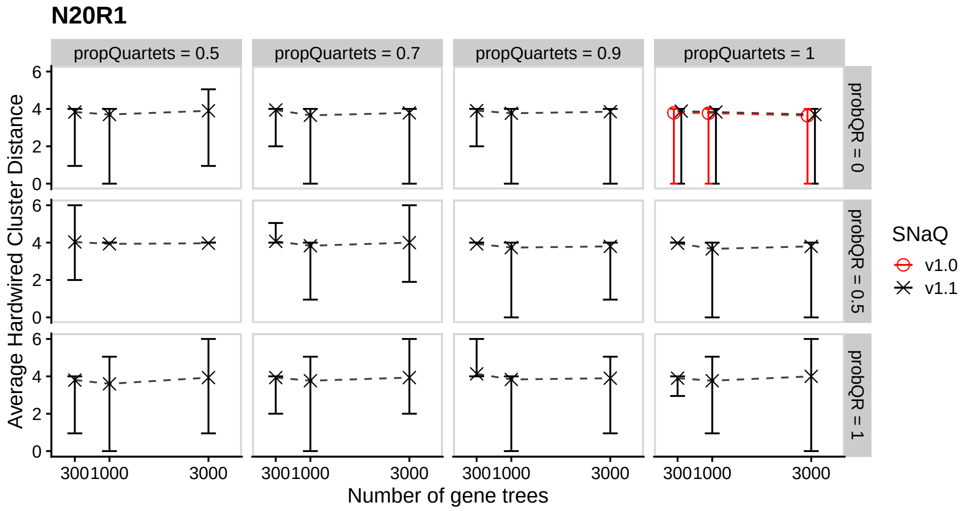


Figure S8: Accuracy results in hardwired cluster distance (HWCD) for the topology with 20 taxa and 1 reticulation relative to the number of input gene trees across a wide array of simulation parameters. Margins represent 95% empirical confidence intervals for observed accuracies, while point estimates are the mean HWCDs for each parameter combination. Simulations utilizing `SNaQ.jl` version 1.0 are shown in red, whereas those utilizing `SNaQ.jl` version 1.1 are shown in blue.

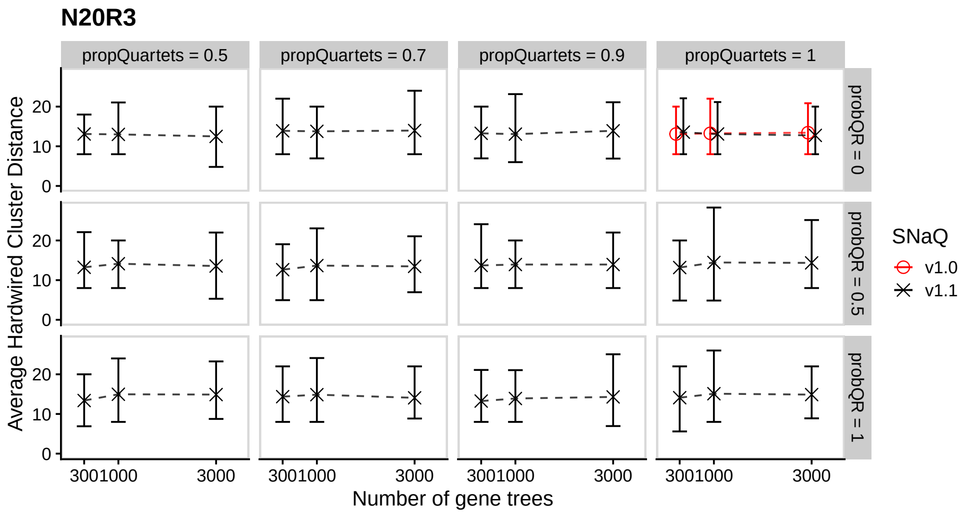


Figure S9: Accuracy results in hardwired cluster distance (HWCD) for the topology with 20 taxa and 3 reticulations relative to the number of input gene trees across a wide array of simulation parameters. Margins represent 95% empirical confidence intervals for observed accuracies, while point estimates are the mean HWCDs for each parameter combination. Simulations utilizing `SNaQ.jl` version 1.0 are shown in red, whereas those utilizing `SNaQ.jl` version 1.1 are shown in blue.

## 1.3 Runtime results

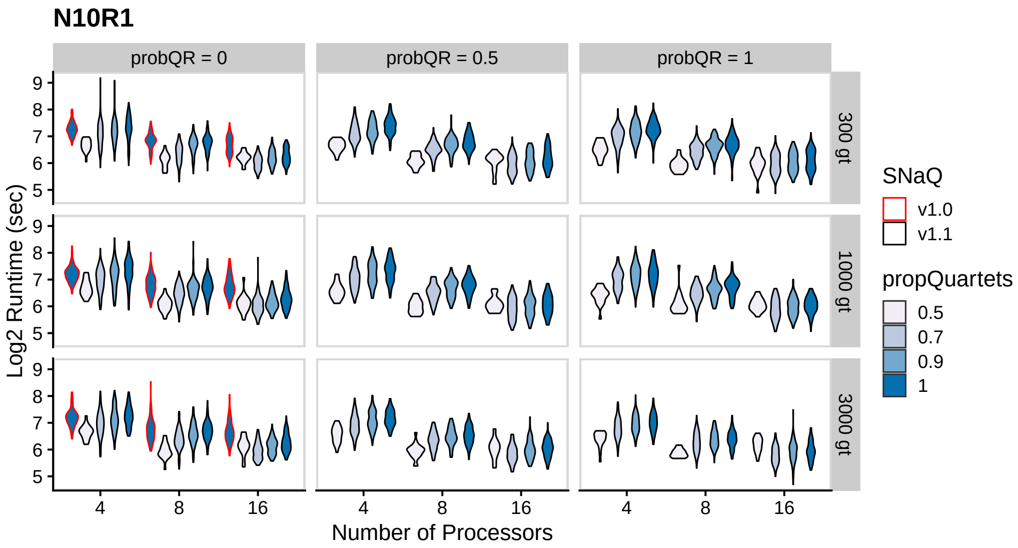


Figure S10: Log2 runtime results in seconds for the topology with 10 taxa and 1 reticulation across a wide array of simulation parameters. Violin plots outlined in red represent results for `SNaQ.jl` version 1.0, whereas all others represent results for version 1.1.

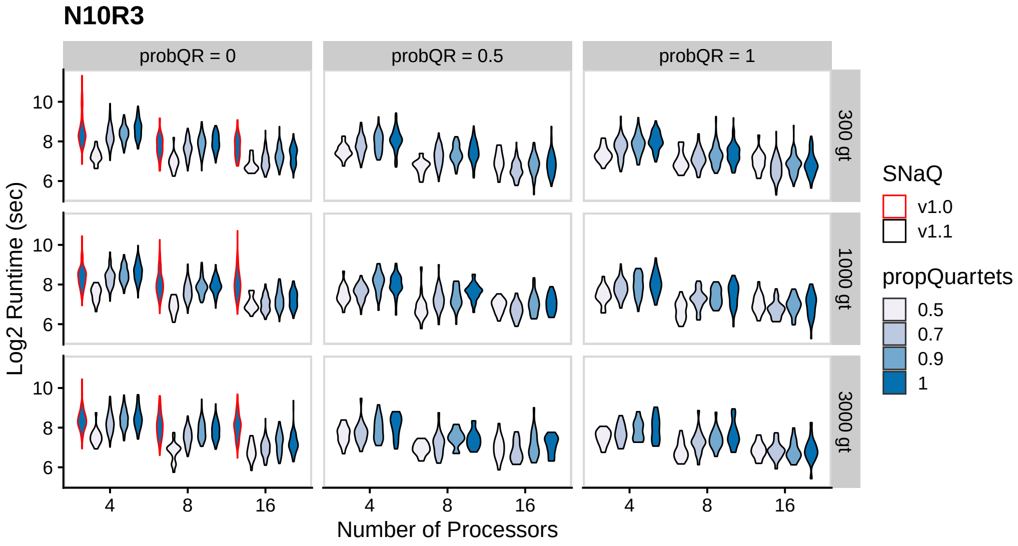


Figure S11: Log2 runtime results in seconds for the topology with 10 taxa and 3 reticulations across a wide array of simulation parameters. Violin plots outlined in red represent results for `SNaQ.jl` version 1.0, whereas all others represent results for version 1.1.

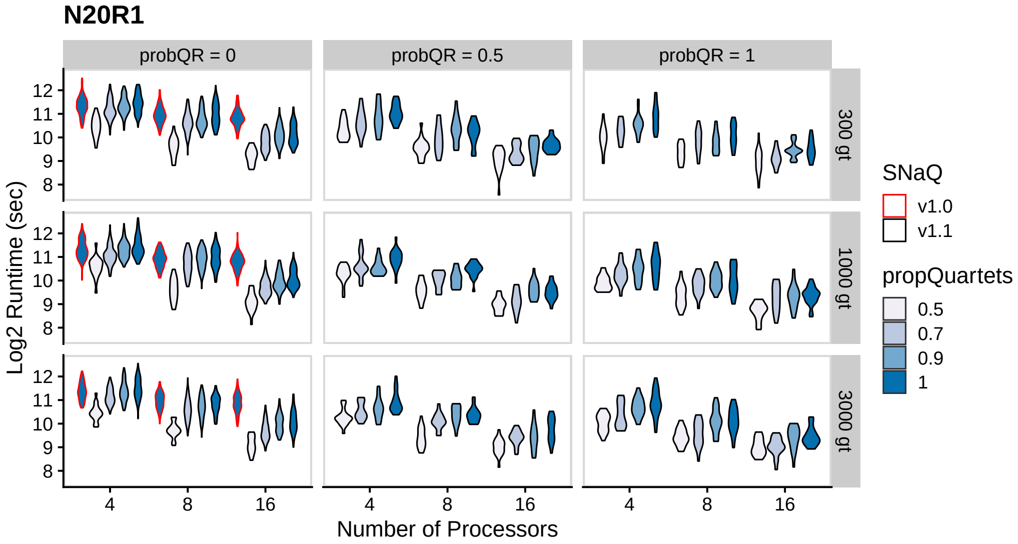


Figure S12: Log2 runtime results in seconds for the topology with 20 taxa and 1 reticulation across a wide array of simulation parameters. Violin plots outlined in red represent results for `SNaQ.jl` version 1.0, whereas all others represent results for version 1.1.

# 2 Empirical Results

| **Runtimes (hours)** | | | | | | |
|---|---|---|---|---|---|---|
| **Reticulation** ($h$) | 0 | 1 | 2 | 3 | 4 | 5 |
| **Version 1.0** | 17.3 | 29.3 | 37.8 | 58.2 | 208.8 | 48.8 |
| **Version 1.1** | 8.0 | 16.5 | 15.8 | 3.2 | 4.4 | 6.1 |
| **Difference** | 9.3 | 12.9 | 22.0 | 55.1 | 204.3 | 42.7 |

Table 1: Runtimes (measured in hours) and improvements from `SNaQ.jl` v1.0 to v1.1 on empirical data with 24 taxa and networks inferred with $h$ reticulations using 10 CPU cores and 2 threads per core. The difference in efficiency measured in hours is also provided.

## 2.1 Inferred networks

Below are all networks topologies inferred for species *Xiphophorus*: Poeciliidae with $h \in \{0, 1, 2, 3, 4, 5\}$ inferred by `SNaQ.jl` versions 1.0 and 1.1 with *X. mayae* arbitrarily chosen as outgroup in all figures.

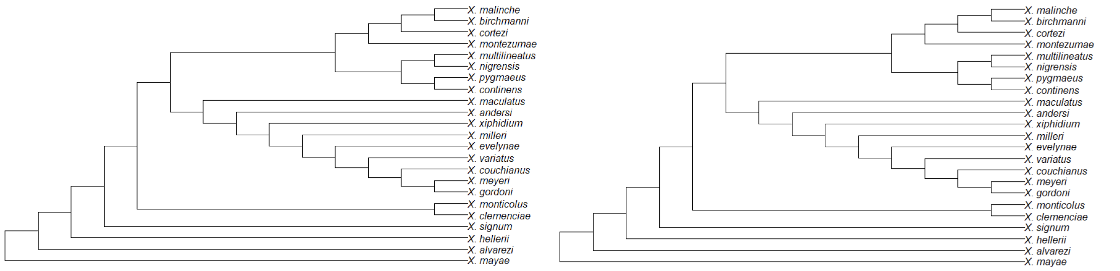


Figure S13: Highest negative log likelihood empirical network inferred by `SNaQ.jl` version 1.1 (left; negative log composite likelihood $\approx 12306$) and version 1.0 (right; negative log composite likelihood $\approx 12306$) with 0 hybridizations.

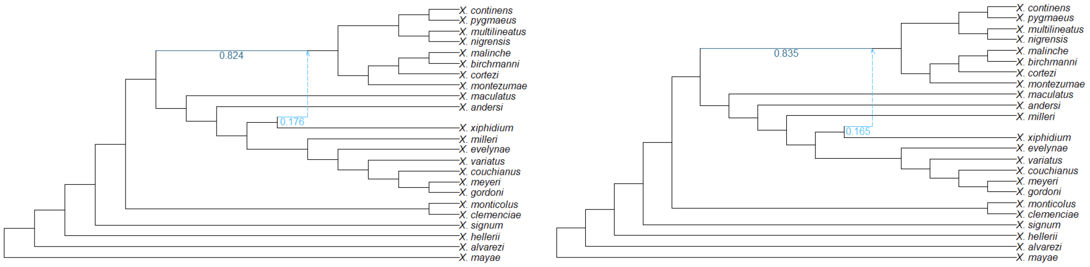


Figure S14: Highest negative log likelihood empirical network inferred by `SNaQ.jl` version 1.1 (left; negative log composite likelihood ≈ 8087) and version 1.0 (right; negative log composite likelihood ≈ 10051) with 1 hybridization. Hybrid inheritance proportions are labelled in cyan font.

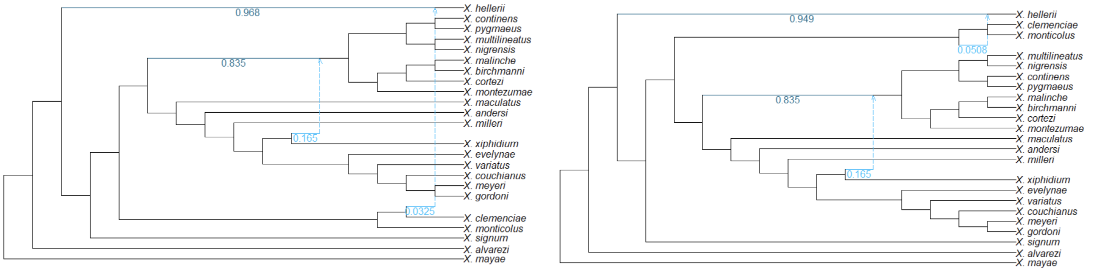


Figure S15: Highest negative log likelihood empirical network inferred by `SNaQ.jl` version 1.1 (left; negative log composite likelihood ≈ 8010) and version 1.0 (right; negative log composite likelihood ≈ 8015) with 2 hybridizations. Hybrid inheritance proportions are labelled in cyan font.

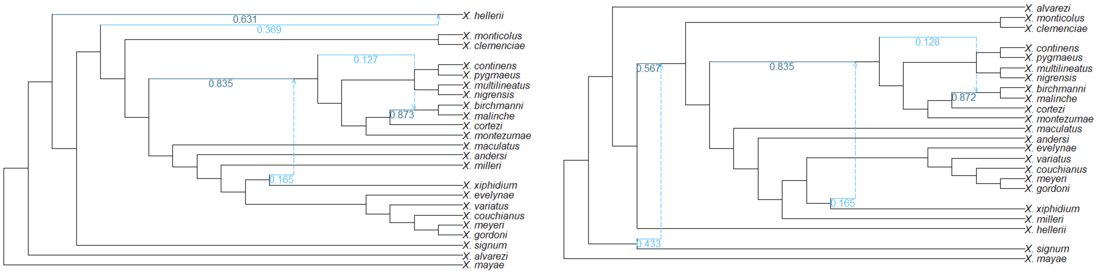


Figure S16: Highest negative log likelihood empirical network inferred by `SNaQ.jl` version 1.1 (left; negative log composite likelihood ≈ 7045) and version 1.0 (right; negative log composite likelihood ≈ 7268) with 3 hybridizations. Hybrid inheritance proportions are labelled in cyan font.

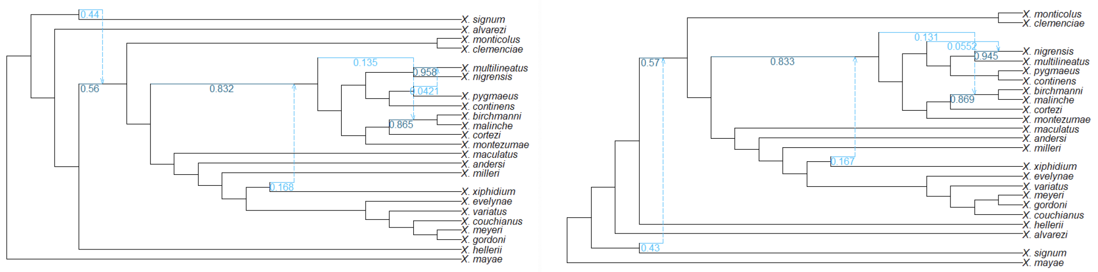


Figure S17: Highest negative log likelihood empirical network inferred by `SNaQ.jl` version 1.1 (left; negative log composite likelihood ≈ 6911) and version 1.0 (right; negative log composite likelihood ≈ 7053) with 4 hybridizations. Hybrid inheritance proportions are labelled in cyan font.

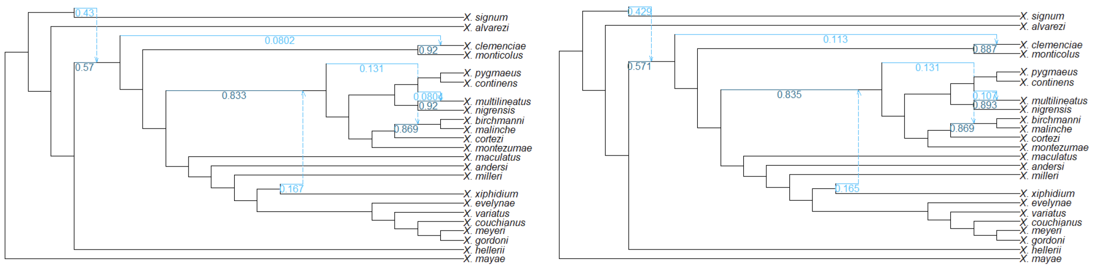


Figure S18: Highest negative log likelihood empirical network inferred by `SNaQ.jl` version 1.1 (left; negative log composite likelihood ≈ 6911) and version 1.0 (right; negative log composite likelihood ≈ 6943) with 5 hybridizations. Hybrid inheritance proportions are labelled in cyan font.

## 2.2 Model selection curves

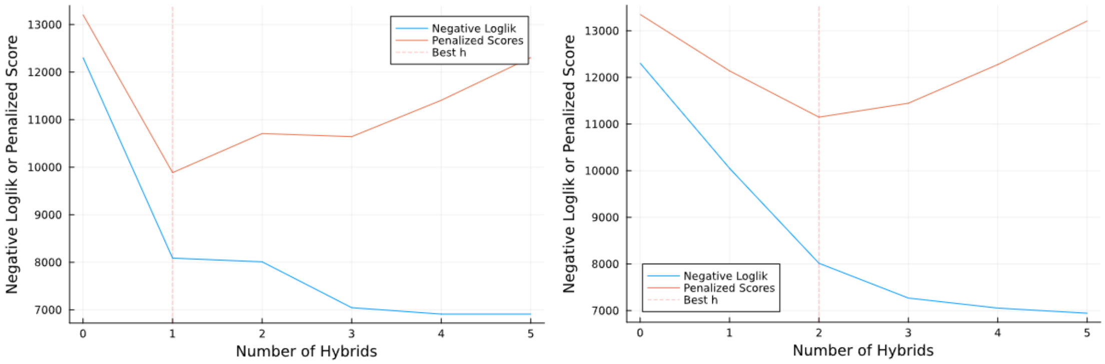


Figure S19: Best inferred log composite likelihoods, slope heuristic penalized scores, and selected model for each empirical network with $h \in \{0, 1, 2, 3, 4, 5\}$ inferred by `SNaQ.jl` version 1.1 (left) and version 1.0 (right).

## 2.3 Networks inferred with $h = 2$ and propQuartets $< 1$

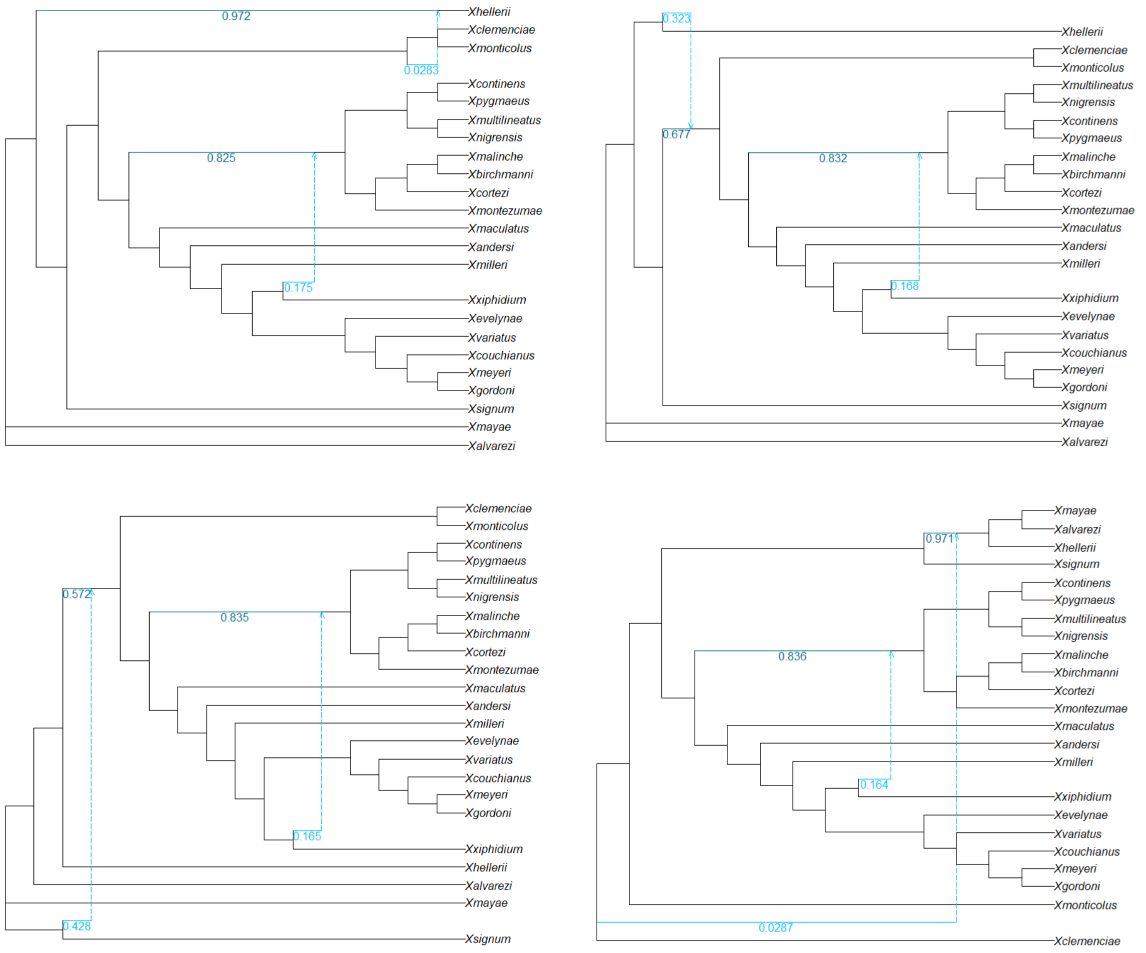


Figure S20: Empirical networks inferred with $h = 2$ and various propQuartets values. **Top left:** propQuartets $= 0.1$ (-loglik $\approx 8080$), **top right:** propQuartets $= 0.3$ (-loglik $\approx 7729$), **bottom left:** propQuartets $= 0.5$ (-loglik $\approx 7663$), **bottom right:** propQuartets $= 0.7$ (-loglik $\approx 8020$).
